# Systems biology of chromium-plant interaction: insights from omics approaches

**DOI:** 10.3389/fpls.2023.1305179

**Published:** 2024-01-08

**Authors:** Abdullah, Kaiser Iqbal Wani, M. Naeem, Prakash Kumar Jha, Uday Chand Jha, Tariq Aftab, P. V. Vara Prasad

**Affiliations:** ^1^ Department of Botany, Aligarh Muslim University, Aligarh, India; ^2^ Department of Plant and Soil Sciences, Mississippi State University, Starkville, MS, United States; ^3^ Indian Institute of Pulses Research (IIPR), Indian Council of Agricultural Research (ICAR), Kanpur, India; ^4^ Department of Agronomy, Kansas State University, Manhattan, KS, United States; ^5^ Department of Agronomy; and Feed the Future Innovation Lab for Collaborative Research on Sustainable Intensification, Kansas State University, Manhattan, KS, United States

**Keywords:** chromium, defense mechanisms, omics approaches, stress tolerance, transporters

## Abstract

Plants are frequently subjected to heavy metal (HM) stress that impedes their growth and productivity. One of the most common harmful trace metals and HM discovered is chromium (Cr). Its contamination continues to increase in the environment due to industrial or anthropogenic activities. Chromium is severely toxic to plant growth and development and acts as a human carcinogen that enters the body by inhaling or taking Cr-contaminated food items. Plants uptake Cr via various transporters, such as sulfate and phosphate transporters. In nature, Cr is found in various valence states, commonly Cr (III) and Cr (VI). Cr (VI) is soil’s most hazardous and pervasive form. Cr elevates reactive oxygen species (ROS) activity, impeding various physiological and metabolic pathways. Plants have evolved various complex defense mechanisms to prevent or tolerate the toxic effects of Cr. These defense mechanisms include absorbing and accumulating Cr in cell organelles such as vacuoles, immobilizing them by forming complexes with organic chelates, and extracting them by using a variety of transporters and ion channels regulated by various signaling cascades and transcription factors. Several defense-related proteins including, metallothioneins, phytochelatins, and glutathione-S-transferases aid in the sequestration of Cr. Moreover, several genes and transcriptional factors, such as *WRKY* and *AP2/ERF* TF genes, play a crucial role in defense against Cr stress. To counter HM-mediated stress stimuli, OMICS approaches, including genomics, proteomics, transcriptomics, and metallomics, have facilitated our understanding to improve Cr stress tolerance in plants. This review discusses the Cr uptake, translocation, and accumulation in plants. Furthermore, it provides a model to unravel the complexities of the Cr-plant interaction utilizing system biology and integrated OMICS approach.

## Introduction

Heavy metals (HMs) contamination is a severe environmental issue on a global scale because it affects soil quality, food safety and food yields, which can have adverse effects on human health (Rai et al., 2019). The lack of planning and regulation in urban and industrial development, pollutants and waste management contributes to environmental pollution (Dabir et al., 2019; Wei et al., 2022). According to the World Health Organization (WHO), exposure to dangerous pollutants like HMs resulted in more than 1.7 million human fatalities (Xu et al., 2018). In the ecosystem at various trophic levels, bioaccumulation and movement of these HMs take place (Pushkar et al., 2021). Chromium (Cr) is one of the HMs that is hazardous to plants, animals, and humans. The extensive use of Cr in industrial and mining activities has led to the release of large amounts of Cr (VI) into the environment, causing a severe environmental problem. (Mehmood et al., 2022). Cr is found in the Earth’s mantle in the 17^th^ highest abundance, and its valence state controls how harmful it is to plants. Many industries, including chemical, mining, steel, tanneries, and Cr plating, employ Cr extensively (Pushkar et al., 2021). Because it is an oxidizing metal, Cr interacts easily with atmospheric oxygen.

Cr can exist in various oxidation states in nature, ranging from 0 to +6. The two most stable forms of Cr in the environment are trivalent and hexavalent (Shahid et al., 2017; Wani et al., 2022). In comparison to Cr (III), Cr (VI) is thought to be more mobile and soluble at all pH levels, which increases its bioavailability and toxicity (Ceballos et al., 2023). Plants lack Cr transporters and absorption pathways, as it is a non-essential element. However, plants can accumulate Cr by using other transporter ions, such as Fe transporters for Cr (III) and phosphate and sulfate transporters for Cr (VI) (Anjum et al., 2016). The capacity of plant roots to absorb vital nutrients was found to be decreased by Cr absorption (Wakeel et al., 2020). Cr causes phytotoxicity upon entering the plant by immediately interacting with it, altering its metabolic pathways, and producing and accumulating reactive oxygen species (ROS), which results in membrane damage (Arif et al., 2019). Excess amounts of ROS can cause cell death by damaging DNA and RNA, inactivating enzymes, oxidizing proteins, and causing lipid peroxidation (Srivastava et al., 2021). Despite this fact, it has been found that Cr (III) is less harmful than Cr (VI), and animals need it to maintain their metabolic processes (Mohan and Pittman Jr, 2006; Urrutia et al., 2008). There is contradictory evidence from different research studies, on whether or not Cr is essential for plant metabolism. While few studies suggest that Cr is not a necessary element for plants, others have shown that small amounts can boost plant growth and yield (Ghosh and Singh, 2005).

Our understanding of the complex and multifaceted interactions between plant metabolism and HMs like Cr has improved as a result of research studies with new and improved omics technologies. In order to analyze the molecular mechanisms behind plants’ stress tolerance to HMs, high-throughput OMICS techniques have been widely used recently. Under conditions of HM stress, plants have evolved coordinated homeostatic systems to control the absorption, mobilization, and intracellular concentration of harmful metal ions in order to reduce the damaging effects of stress. Omic studies give us a more detailed image of the networks and pathways that are principally responsible for cellular detoxification and the tolerance mechanism against HM toxicity. It may be possible to adapt recent omics discoveries into a dependable, efficient, and ecologically friendly technology. In this regard, it is imperative that “Omics” studies—which include proteomics, metallomics, and genomics—be taken into account for the sake of the improvement and selection processes.

The advancements in research on Cr uptake and effects inside plants is the main focus of this review. To close the knowledge gap concerning gene expression regarding the fate of Cr inside the plants, we compiled pertinent research about the underlying mechanism of Cr uptake and insights on molecular aspects. This review will aid in advancing investigations into the complex relationship between Cr and plants. Furthermore, understanding the mechanisms at the molecular level of Cr-plant interactions and its biogeochemistry can provide new insights into how it could be managed by chemical, microbiological, and genetic methods, which will protect crop production, crop yields and agricultural sustainability. Additionally, the accumulation of Cr in crops grown on contaminated soils causes substantial risks to human and animal health. The growth of long-lasting remediation techniques and a complete understanding of Cr’s biogeochemical activity in soil is thus required for its alleviation. An extensive review of the available knowledge shows that the present evidence in the study of HM plant interactions provides the ability to comprehend the HM-related changes in plants through OMICS methodologies, which we can combine with other methods to minimize the negative impacts of Cr on plants. Additionally, OMICS techniques can be used to develop HM-tolerant crop varieties using targeted breeding programs.

## Sources and occurrence of chromium

The Cr containing products are widely used in many industrial and agricultural operations. Due to this, its pollution has grown to be a significant issue in the environment. Environmental Cr toxicity results from both man-made and natural sources. Groundwater is a natural source of chromium, and its dominant form in groundwater is Cr (VI). It is presumed that Cr (VI) is derived from the leaching of minerals. However, Cr pollution in the environment is primarily caused by anthropogenic activities, such as paper and pulp mill effluent streams, the leather tanning industry, non-ferrous base metal smelter refineries, discharges from thermal power generating stations, and urban stormwater runoff (World Health Organization, 2020). Cr is released from the tanning industry, which utilizes Cr_2_(SO_4_)_2_ as a tanning agent but discards unused 40% of it into the environment (Rahman, 2018).

The concentration of Cr in the soil impacts plants’ physiology, development activity and nutrient uptake. In plants, Cr (III) is a stable form. At the same time, Cr (VI) is the phytotoxic form where they are present as a compound like the reaction between Cr (VI) and oxygen gives chromate (CrO_4_^2-^) or dichromate (Cr_2_O_7_^2-^), whereas Cr (III) exists as chromite (FeOCr_2_O_3_) (Ertani et al., 2017; Wani et al., 2022). Cr accumulates many anions and causes the soil pH to rise, due to which dehydrogenase activity and alkaline phosphatase are suppressed in the soil due to Cr slag (Liu et al., 2019).

## Chromium uptake

The entire plant Cr uptake process is poorly understood, but as a nonessential element, it lacks any particular mechanism. However, Cr (III) is taken up passively, that is, without any energy consumption (Ao et al., 2023). Cr (VI) shares structural similarities with sulfate and phosphate, so its absorption occurs via phosphate and sulfate transporters by an energetically dependent active mechanism (Ding et al., 2019). Therefore, metal speciation, which determines its absorption, translocation, and accumulation, determines its uptake. The solubility of Cr (VI) is higher than Cr (III); thus, it is more toxic at lower concentrations than the latter and has a propensity to form more persistent complexes in soil (Abdulmalik et al., 2023). The absorption and translocation of Cr (VI) have produced inconsistent results; some authors argue that Cr (VI) is converted to Cr (III) on the surface of the roots (Wani et al., 2022), while others postulate that plants can absorb dissolved Cr (VI) without reduction (Malaviya and Singh, 2011).

## Chromium accumulation and translocation

The accumulation and translocation of Cr is plant species dependent and varies with the oxidation state and concentration of Cr present in the growth media or soil (Shahid et al., 2017). Its concentration in roots is reportedly much higher than in shoots due to its low mobility in the plants (Gupta and Sinha, 2006). Various researchers have investigated the cause of its low mobility (Karimah et al., 2021). Cr follows Michaelis-Menten kinetics at low concentrations because sulfate competitively inhibits it (Skeffington et al., 1976), as the sulfate concentration in the soil is relatively high. Poor translocation of Cr to the shoots may be caused by the majority of Cr being sequestered into the root cell’s vacuoles to render it non-toxic, which is a defensive approach of the plant (Shanker et al., 2004). Its toxicity also results in differential uptake and distribution of various other metal elements. For instance, Cr increases zinc (Zn) concentration in the roots, stem, and seeds, but in leaves, its concentration decreases, whereas iron (Fe) concentration decreases in the stem, leaves, and seeds and increases in roots (Tiwari et al., 2009). This Fe deficiency and restriction of Fe from root to shoot suggests that Fe can be displaced from physiologically active sites by Cr (Chatterjee and Chatterjee, 2000). Thus, it is understood that the translocation of Cr is poor from root to shoot and follows an order of root > stem > leaves > seeds (Tiwari et al., 2009). It has also been suggested that Cr (III) can bind with cell walls in plant tissues, further restricting its translocation (Kabata-Pendias and Szteke, 2015).

## Impact of chromium on plants

Cr toxicity has been shown to have a negative impact on a number of elements of plant biology, including growth, physiology, development, and crop output. According to Rath et al. (2019), Cr toxicity causes a number of physiological changes in plants, such as impaired seed germination, decreased plant growth and development, reduced leaf area, decreased photosynthetic activity, altered cell membrane characteristics, leaf chlorosis, and damage to cell walls. Cr interferes with the absorption and utilization of vital mineral elements, which is one of the main negative effects it has on plant physiology. It competes with the uptake of some essential minerals including Ca and Fe, which causes an imbalance in nutritional levels. This disruption in nutrient absorption manifests as symptoms of nutrient deficiency, resulting in stunted growth and decreased photosynthetic efficiency, ultimately impacting plant growth and crop yield (Kotaś and Stasicka, 2000). It has been observed that *Vigna radiata* plants grown in soil contaminated with 50% Cr exhibited a significant decrease in their amino acid, starch, and chlorophyll levels (Rath et al., 2019). Furthermore, another study by Sundaramoorthy et al. (2010) reported that Cr inhibits the process of mitotic cell division in *Oryza. sativa* seedlings subjected to Cr stress, leading to a delayed and prolonged cell cycle. Additionally, Cr induces oxidative stress within plants by generating ROS such as superoxide radicals and hydrogen peroxide. These ROS can cause damage to vital cellular components like proteins, lipids, and DNA, disrupting normal cellular functions, impairing metabolism, and retarding the growth and development of plants (Shanker et al., 2005).

Roots serve as the primary interface between plants and soil contaminated with Cr. Research studies have shown a notable reduction in root elongation, root diameter, and root hair growth in conjunction with root dieback when plants are exposed to elevated levels of Cr (Zulfiqar et al., 2023). High concentrations of Cr can lead to structural alterations in roots. This compromised root system restricts the plant’s capacity to absorb both water and essential nutrients from the soil, consequently impeding overall growth and crop yield (Shahid et al., 2017). Moreover, Cr-induced stress disrupts the efficient allocation of resources within the plant, resulting in diminished leaf size, reduced flower production, and lower yields of fruits or seeds (Shanker et al., 2005).

To alleviate the detrimental effects of Cr on plants, it is imperative to attain a comprehensive understanding of the underlying molecular mechanisms involved. Cutting-edge techniques within the field of ‘omics,’ including genomics, transcriptomics, proteomics, metabolomics, and ionomics, hold the potential to provide valuable insights into how plants respond to Cr-induced stress. By pinpointing key genes, proteins, and metabolites associated with detoxification and tolerance mechanisms, researchers can formulate strategies aimed at bolstering a plant’s ability to withstand Cr pollution. The application of ‘omics’ approaches has great promise in unraveling the intricate dynamics of Cr-plant interactions and in developing effective measures to mitigate the harm caused by Cr to agricultural ecosystems.

## Recent advancements in plant responses to chromium stress (omics approach)

**Figure 1:**
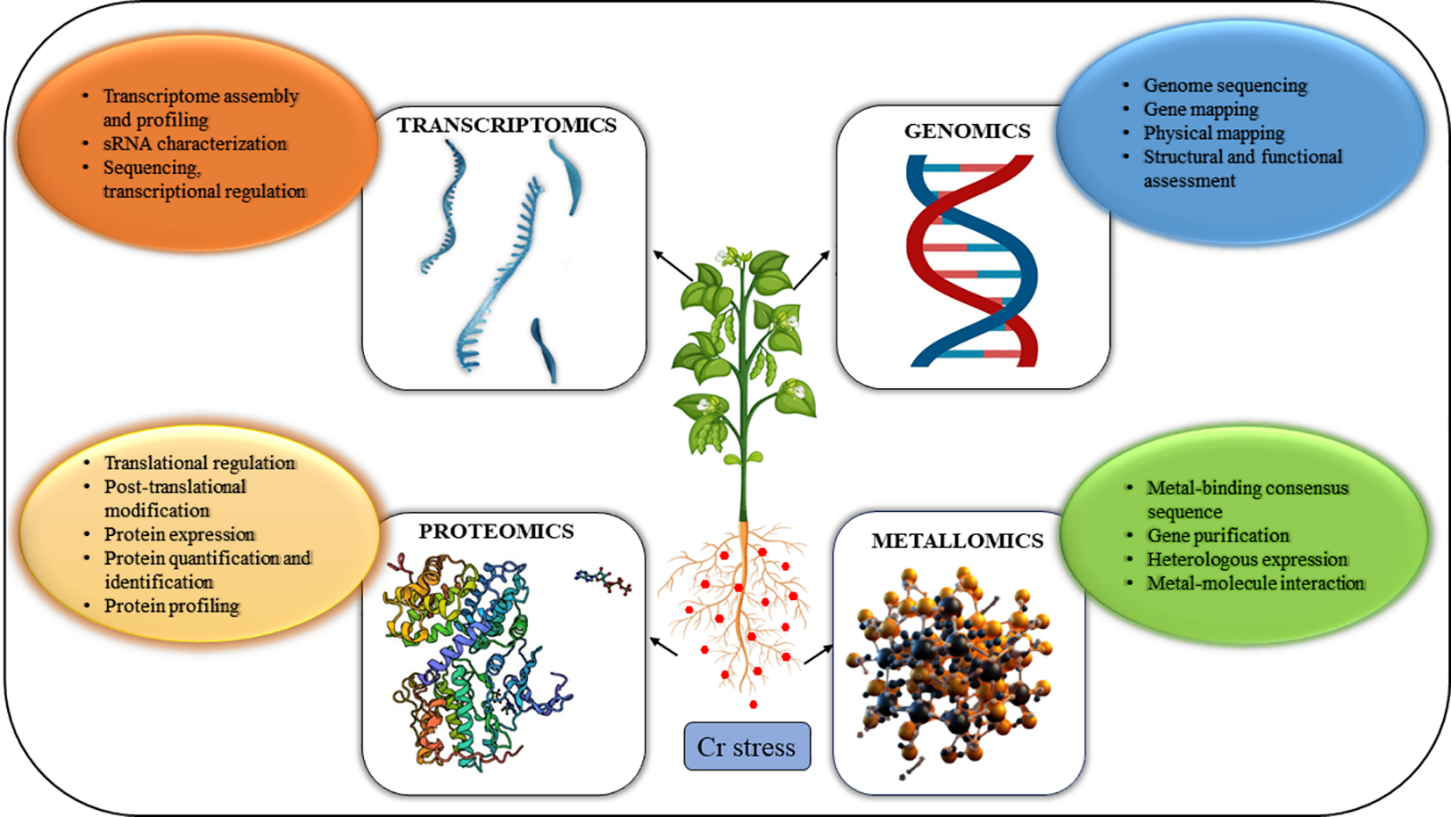
Combined OMICS strategies to combat the toxicity of HMs in plants are shown schematically. Integrated Omic approaches such as genomics, transcriptomics, proteomics, and metallomics have a wide scope to combat heavy metal stress in plants. These high throughput techniques contribute to the comprehensive analysis of genes, regulators, proteins, and signaling networks that facilitate advancement in plant stress responses and crop improvement.

Although numerous studies have been conducted on the effects of Cr stress, the molecular mechanisms underlying Cr phytotoxicity, plant defense mechanisms, and transportation and accumulation in plants remain unclear (Wani et al., 2022). However, due to recent developments in the “omics” disciplines, investigations in this area may now be carried out with much better precision and analysis of a more significant number of variables associated with physio-molecular responses to stress from Cr as depicted in **Figure 1**. Furthermore, “omics” disciplines have enormous potential to understand the mechanisms that underlie the toxicological impacts of chemical contaminants, and as a result, leading to the discovery of novel biomarkers which are impacted by stress and can be used as screening tools to identify tolerant genotypes.

## Biparental QTL mapping for chromium tolerance in plants

Advances in genomics resources in various crop plants have greatly facilitated the uncovering of multiple QTLs controlling HM tolerance using a bi-parental mapping population (Qiu et al., 2010; Qiu et al., 2011). QTL mapping, as a popular and effective method for genetic analysis, has yielded numerous notable results in the field of metal stress tolerance in plants (Raj and Nadarajah, 2022; Hassan et al., 2023). However, very limited studies have been carried out about the genetic analysis of QTL related to Cr accumulation and tolerance in plants. Aiming to elucidate QTLs maintaining Cr tolerance in *O. sativa*, three QTLs preventing Cr accumulation were identified from a double haploid mapping population in *O. sativa* (Qiu et al., 2010). The *qRCA7* QTL contributed to Cr accumulation, explaining 21.3% phenotypic variation (PV) on Chromosome 7, and *qSCA QTL* attributed to shooting Cr accumulation, explaining 22.7% phenotypic variation (PV) on Chromosome 9 was uncovered. Another QTL *qSRA7 located* on chromosome 7 and responsible for the ratio of shoot Cr accumulation (SCR) and root Cr accumulation (RCA), explaining 12.7% PV, was also elucidated (Qiu et al., 2010). Further, Qiu et al. (2011) uncovered another two major QTLs, *qSCC10*, existing on chromosome 10, explaining >10% PV, and *qSRC10*, explaining 11% PV and overlap with the *qSCC10* QTL on chromosome 10 from double haploid mapping population. However, the number of Cr-tolerant QTLs reported in *O. sativa* and other crops remain limited.

## Functional genomics studies

Recent investigations through genomic technologies have improved our understanding of how plants cope with Cr stress by examining myriads of gene expression at a time. The expression profiles were analyzed using a microarray test to demonstrate how *O. sativa* roots responded to Cr (VI) stress (Huang et al., 2014). Around 2688 genes were found to be responsive to Cr and participate in binding activity, metabolism, biological control, catalysis, and cellular function. Exposure to Cr for a shorter period (1-hour and 3-hour combined, 1181 genes) resulted in fewer transcripts associated with Cr than exposure for a more extended period (24 hours, 2097 genes). This suggests that the exposure period to Cr may affect transcriptome profiles (Huang et al., 2014). Additionally, short-term Cr exposure resulted in the upregulation of many kinases.

Another study observed that the endonuclease attack and multi-copy transposition on DNA are averted by high methylation levels, which favors HM tolerance in *O. sativa* (Feng et al., 2016). Based on data from earlier research, it appears possible that DNA methylation regulates plant reactions to HMs via two different pathways (Arif et al., 2016). The first mechanism has to do with methylation’s ability to shield DNA against HM-induced single-strand breaks or multi-copy transposition. Gullì et al. (2018) found, that the genomes of *Noccaea caerulescens* plants-a Ni hyperaccumulator species—grown in high Ni doses were substantially more hypermethylated than those of *A. thaliana* plants, which are susceptible to high Ni doses. Controlling gene expression is a second kind of epigenetic response to HM stressors. This control extends to the coding regions of genes as well as their promoter regions. Oono et al. (2016) showed a positive relationship between the Cd dose response in plants and the expression of genes coding metal ion transporters where DNA methylation markers were found.

Using MSAP and immunolabelling techniques, Cr modifies the methylation level of the rape genome, and levels of hypermethylation and the stress dose of Cr correlated well (Yang et al., 2007). Contrarily, some research has shown that Cr stress lowers cytosine methylation levels by 20–40% in clover and industrial hemp (Aina et al., 2004; Shaikh et al., 2022). These differences showed that various plant species have specific methylation processes for Cr resistance (Peng and Zhang, 2009).

Micro RNAs (miRNAs) regulate HM tolerance in plants, including Cr (Mendoza-Soto et al., 2012). miRNAs could be the next target for enhancing plant adaptability and stress tolerance by boosting gene expression that encodes different transcription agents and defense-associated proteins (Ghosh et al., 2022). Understanding the differences in miRNA and their related target genes under Cr (VI) stress in *O. sativa, Nicotiana tabacum*, and *Raphanus sativus* has only been addressed in a few isolated studies (Bukhari et al., 2015; Liu et al., 2015; Dubey et al., 2020). In tobacco plants, a comparative genomic study on 41 conserved Cr-responsive miRNA families revealed that, under Cr stress, around 11 families of miRNA were found to be increased only in species that are Cr-tolerant but remained unchanged in Cr-sensitive ones, whereas 17 families of miRNA were found to be increased in the species that are Cr-sensitive (Bukhari et al., 2015). Only a single family, miR6149, was downregulated in the Cr-sensitive genotype but remains unaltered in Cr-tolerant ones. In the two genotypes under Cr stress, 14 of the 29 newly discovered distinct miRNA families expressed differentially, providing crucial information on the function of miRNAs in Cr tolerance (Bukhari et al., 2015). Similarly, several well-conserved up- or down-regulated miRNAs were discovered in *O. sativa*; in response to short-term Cr stress, six miRNA families were up-regulated, and six were down-regulated. The expression of only three miRNAs (osa-miR5072, osa-miR444, and osa-miR396) was suppressed by continuous stress, whereas only two miRNAs (osa-miR166 and osamiR171) were upregulated (Dubey et al., 2020).

## Transcriptomic studies

Transcriptome profile analysis (TPA) provides novel insights for identifying the numerous genes associated with Cr stress (Nie et al., 2021). Advent of the next-generation sequencing (NGS) method has recently made gene expression profiling, transcriptome assembly, and RNA sequencing more effective (Satam et al., 2023). Thus, allowing us to examine an organism’s gene activity at various stages under varied circumstances (Geng et al., 2011). These methods disclosed some crucial genes encoding glutathione S-transferase (GSTU6), multidrug resistance protein 4, and PDR-like ABC transporter involved under Cr stress in *O. sativa* (Dubey et al., 2010). Likewise, several differentially expressed genes (DEG) have been identified in radish under Cr stress by Xie et al. (2015). Some of the DEGs like *MAO* (*monoamine oxidase*) and *UGT* (*glucuronosyl transferase*) which were down-regulated under Cr stress, work in a pathway like drugs and xenobiotics metabolism and serine and threonine metabolism respectively. Moreover, *GST* (*glutathione S-transferase*) and *AGXT* (*serine-pyruvate transaminase*) were up-regulated and work in the same pathway as *MAO* and *UGT*. This suggested that certain genes might interact with one another to do specialized functions in particular biological processes (Xie et al., 2015). Calcium-binding protein genes and calcium-dependent protein kinases like *CML42, CML44*, and *CML50*, were up-regulated under Cr stress in radish roots (Xie et al., 2015. *De novo* transcriptome assembly of radish root treated with Cr stress allowed the identification of 30,676 unigenes representing 60,881 transcripts. Substantial differences in the expression of 2,985 unigenes from Cr-free and Cr-treated libraries were noted. Among these genes, 1424 were upregulated and 1,561 were down-regulated (Xie et al., 2015). In radish, some miRNA families (miR156/157, miR159, and miR5293) play a crucial role in Cr homeostasis by targeting SPLs (SQUAMOSA promoter-binding protein-like) that are found to be involved in different development processes like flowering (Yu et al., 2012), shoot growth (Nonogaki, 2010) and metal homeostasis (Pilon et al., 2009).

In one of the recent findings in germinating seedlings in soybean (Chen et al., 2023), transcriptome sequencing yielded a total of 13,777 differentially expressed genes (DEGs), and weighted correlation network analysis (WGCNA) revealed that 1298 DEGs across six gene modules had a strong connection with physiological characteristics. It has been suggested that the DEGs encoding antioxidant enzymes, ion transporters, and transcription factors provide Cr tolerance in soybean germinating seedlings by lowering the level of ROS, preventing Cr uptake and translocation, and maintaining the osmotic balance in germinating seedlings. Cr-induced ROS activates MAPKs, which are involved in the signal transduction in radish by regulating the transcription factors (Sanchita et al., 2014). Eight DEGs are homologous to genes that encode MAPKs in which MAPK18, MAPK20, and other calcium-binding-related protein genes express themselves differently (Wang Y. et al., 2013).

## Proteomic analysis

Proteomics allows us to study the dynamic changes of entire proteins of an organism in response to a particular stress, including HM stress in plants (Yadav et al., 2021). In one of the studies, maize (*Zea mays* L.) plant leaves were subjected to a short time Cr (VI) stress for 1, 6, and 24 hours. A total of 1200 spots were found using two-dimensional electrophoresis (2-DE), of which 60 spots were found to accumulate differentially under Cr stress. Of the Cr-regulated proteins, 58 were identified using tandem mass spectrometry (MS/MS). These controlled proteins are primarily engaged in protein folding and synthesis, processing of rRNA and mRNA, photosynthesis and chloroplast organization, ROS detoxification and defense responses, cytoskeleton, and DNA damage response (Wang R. et al., 2013).

The protein level alterations were assessed in one study using the MALDI-TOF assay after *Pseudevernia furfuracea* was exposed to Cr stress (Özenoğlu-Aydınoğlu et al., 2021). Roughly nine common proteins were found, of which six had up-regulated expression levels (HSP 60, BNI5, VSP64, KIP3, OP4 and BCK1) and three had down-regulated expression levels (MNS1, ATG4, and ABZ2). These proteins were shown to be useful in biological processes including transcription regulation, cellular detoxification metabolisms, and stress signaling. Moreover, HSP60 protein was examined using a western blot technique to verify the degree of protein expression. In comparison to the control sample, it has been demonstrated that *P. furfuracea* exposed to Cr (VI) increased the quantity of HSP60 protein (Özenoğlu-Aydınoğlu et al., 2021). Similarly, a comparative proteomic technique was done between two cultivars of *Brassica napus* grown hydroponically (Cr-sensitive Sary and Cr-tolerant NK Petrol) to examine the variations in protein abundance under S deficiency and Cr (VI) stress. Two-dimensional gel electrophoresis (2-DE) protein pattern analysis showed that 58 protein spots were controlled differentially by Cr (VI) stress (+S/+Cr), combined stress (−S/+Cr), and S-deficiency (−S/−Cr). Thirty-nine protein spots were determined using MALDI TOF/TOF spectrometry. Differentially regulated or controlled proteins were primarily involved in protein folding, stress defense, energy metabolism and stabilization, sulfur metabolism, redox regulation, signal transduction, and photosynthesis. Six stress defense-associated proteins, including glutathione S-transferase, 2-Cys peroxiredoxin BAS1, L-ascorbate peroxidase, ferritin-1, thiazole biosynthetic enzyme, and myrosinase-binding protein such as At3g16470 show a substantial increase in NK Petrol. Under changing S conditions, stress-related proteins are crucial for detoxifying Cr (VI) and preserving the homeostasis of cells (Yıldız and Terzi, 2016).

In a similar study, two varieties of sunflower (*Helianthus annus*; AHO-33 and RA-713) were exposed to varying levels of Cr (control, 200 ppm, and 400 ppm). Applying SDS-PAGE to study plant proteomic modulation revealed that three bands, 60, 40, and 42 kDa, and two protein bands, 49 and 13 kDa, were upregulated in the seeds of AHO-33 and RA-713, respectively. New bands (48, 49, and 26 kDa) had also appeared. In the leaf tissues of both varieties, a few proteins (52, 16 kDa) had their expression downregulated. However, only the six and 81 kDa proteins displayed up-regulation, and the 154 kDa protein showed down-regulation in the shoot. This demonstrates how plants change the protein expression pattern in response to Cr stress (Sardar et al., 2022). Another study used two *O. sativa* genotypes with different Cr tolerance and accumulation levels to examine how the proteome will respond to Cr (VI) stress. Proteomic studies show that *O. sativa* proteome response to Cr stress depends on the tissue, the dosage of Cr, and the genotype. Sixty-four proteins that exhibit differences of at least a factor of four under either of two Cr levels have been successfully found. They participate in various cellular functions, such as the synthesis of cell walls, production of energy, primary metabolism, transport of electrons, and detoxification (Zeng et al., 2014).

## Metallomics approach

Metallomics includes qualitative and quantitative analysis of metals, which aids in building applications for novel techniques in soils with metal contamination (Singh and Verma, 2018; Mahawar et al., 2023). A significant proportion of molecules involved in cellular metabolism and behavior are biomolecules that bind to metals, and understanding the functional roles of a protein’s metal cofactor can help us understand the pathways of cells (Haraguchi, 2017). These metal-binding proteins are environmental biomarkers (López-Barea and Gómez-Ariza, 2006). Studies related to metallomics are still lacking, which may be due to the complexity of the matrices or the low concentration of trace elements in plant tissues (Gómez-Ariza et al., 2004). However, with the advancements of techniques like X-ray absorption spectroscopy (XAS), mass spectrometry (MS), X-ray fluorescence (XRF), proteomic approaches like 2D electrophoresis or different types of chromatography (affinity/liquid), and sensitive techniques like (ESI-MS/ICP-MS) are used that led to new prospects in this area of study (Shi and Chance, 2008).

In relation to Cr, the precise and quantitative measurement of Cr content in plants can be achieved with the help of micro-proton-induced X-ray emission (μ-PIXE) technology (Kachenko et al., 2008; van Der Ent et al., 2018). X-ray absorption near edge structure (XANES) technique allow us to study the interconversion of Cr (VI) to Cr (III) in roots. Furthermore, information regarding potential modifications in the oxidation of Cr atoms attached to the biomass was obtained using XANES. The XANES analysis revealed that the oxygen atoms in the samples were arranged in an octahedral pattern around the core Cr (III) atom (Bluskov et al., 2005). The coordination surroundings, the closest neighboring atoms, and the ligands involved in Cr binding by saltbush biomass were all revealed by Sawalha et al. using extended X-ray absorption fine structure (EXAFS) (Sawalha et al., 2005), Cr localization site and its sequestration in cortical and epidermal cells in roots and spongy mesophyll and epidermal cells in leaves is shown by X-Ray microprobe. In addition to knowing the specific metal or metalloid present in a plant, it is also essential to understand the biomolecules to which it is bound, the coordination groups involved, and its speciation (Arruda and Azevedo, 2009). In this context, different oxidation states of Cr have been studied in Cr (VI) treated wood samples (beech and pine) by applying X-ray absorption near-edge structure (XANES), while with heat exposure, Cr (VI) was wholly reduced to Cr (III) (Strub et al., 2008; da Conceicao Gomes et al., 2017).

## Reactive oxygen species-induced stress and its impact on signaling cascades

**Figure 2:**
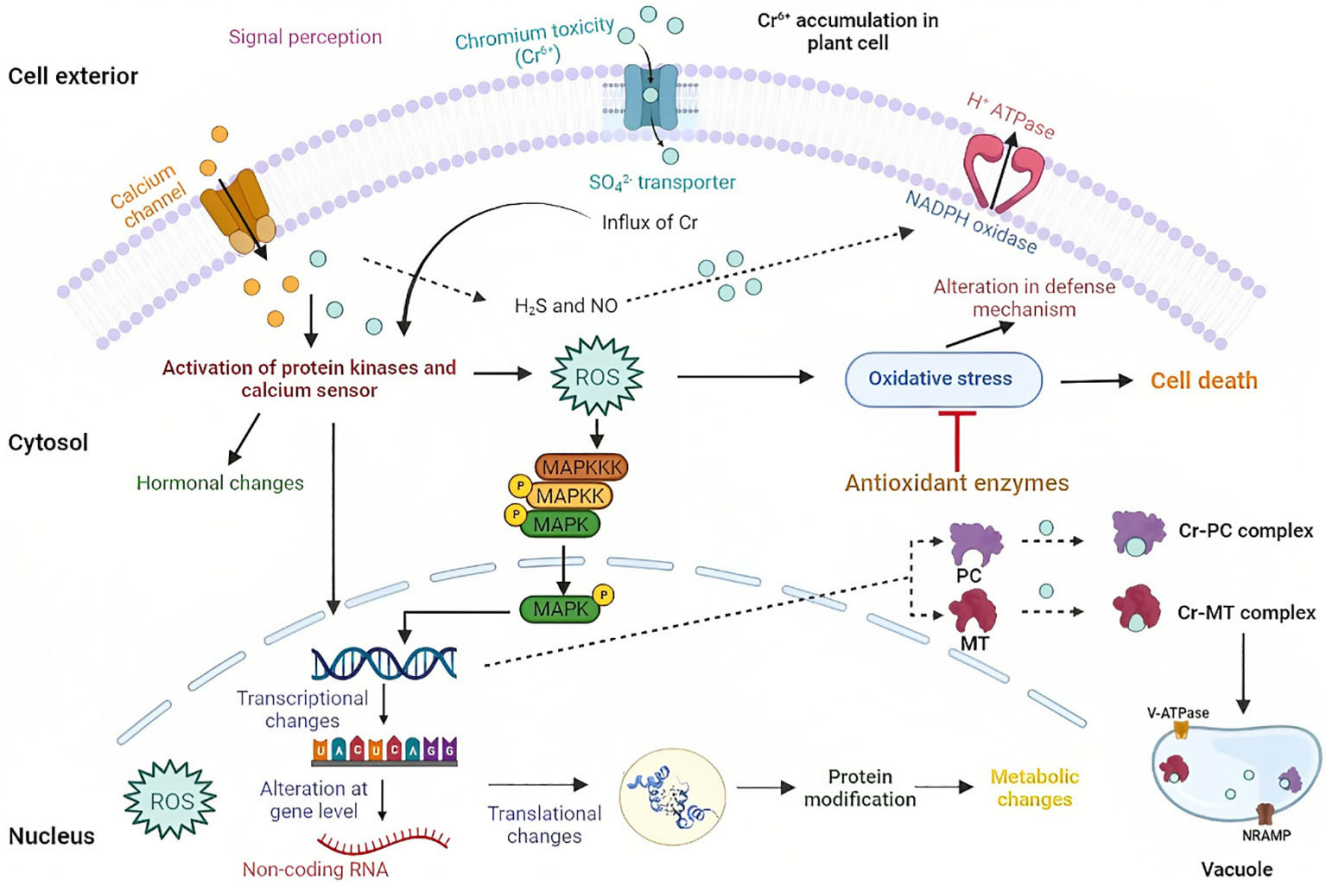
Model illustration showing Cr uptake and accumulation in plant cells. Here, Cr uptake is done through different transporters. Different sensors perceive Cr toxicity, such as receptor-like kinases and channels. After Cr sensing, ROS is generated, which is sensed by different sensors like protein kinases and calcium sensor that makes changes at transcriptional, translational, and genomic levels.

Plants orchestrate various adaptive mechanisms to withstand the challenging and toxic impacts of Cr-HM (Azeez et al., 2021). When exposed to stressful environments, plants produce ROS, a defense mechanism (Gielen et al., 2017). The overabundance of ROS causes endogenous stress that may adversely affect plant growth and development (Wakeel et al., 2019). These are produced in plants as a result of the Haber-Weiss and Fenton reactions by redox metals like Cr, which causes oxidative damage (Flora, 2009) that leads to protein, DNA, pigment damage, and lipid peroxidation (Choudhury and Panda, 2005). In response to HM toxicity, this process is thought to be one of the main factors causing changes in plant biology at the biochemical level (Sharma et al., 2020). The active sites of the enzymes are deactivated when Cr reacts with the protein’s catalytic site by binding to a specific functional group; thus, it changes the activity of enzymes (Gupta et al., 2010). There are still a lot of unraveled molecular mechanisms of Cr signaling from the interior (plasma membrane and cytosol) and exterior (cell wall). Based on available data, this review presents a model describing the Cr signaling cascade in plants, as shown in **Figure 2**.

However, with the development of multiomics, few studies have lately demonstrated metabolic, translational, and transcriptional reprogramming in different plant systems subjected to Cr exposure, offering a unique understanding of Cr sensing and signaling. For instance, in *O*. *sativa*, the presence of Cr (VI) induces ROS and Ca^2+^, followed by the activation of calcium-dependent protein kinase and NADPH oxidase, which are essential for the subsequent signaling process (Trinh et al., 2014). NADPH (nicotinamide adenine dinucleotide phosphate hydrogen) oxidase in the plasma membrane also causes oxidative stress since it is related to Cr (Pourrut et al., 2013). Several defensive signaling cascades, including hormonal (primarily auxin, cytokinins, and ethylene), calcium, and MAPK signaling, are also stimulated by HM stress (Sun et al., 2010). There is growing proof that the ROS and calcium signaling systems interact, which has significant implications for optimizing cellular signaling networks. Many transcription factors, including the *WRKY* and *AP2/ERF* TF genes, are involved in Cr signaling cascades, which strengthened the case for their function in metal stress resistance. Similarly, most phosphate kinase genes (*PP2C-A*, *PP2C-D*, and *PP2C-F*) were found in response to Cr (VI) stress, proving that they might be involved in controlling different signaling processes during Cr stress. Prior gene expression profiling of *O. sativa* under Cr stress indicated the inactivation of ethylene (ET), abscisic acid (ABA), jasmonate-mediated signaling cascades, and the silencing of gibberellic acid-related pathways (Trinh et al., 2014).

Along with ROS production, Cr also activates the signaling of antioxidant defenses, defense proteins like glutathione-S-transferases (GSTs), metallothionine (MTs), and phytochelatins (PCs), which is followed by phytosequestration and compartmentalization (Yu et al., 2019). PCs and MTs are crucial in the detoxification and homeostasis of Cr. Furthermore, they make a complex with the HM, making them non-toxic and transported to vacuoles (Wu et al., 2013; Yu et al., 2019). Increased PC synthesis and glutathione generation were recorded upon Cr (VI) exposure of the *Arabidopsis* plant treated with H_2_S (Fang et al., 2016).

In one of the studies on *O. sativa*, it was observed that with the HM stress, the activity of CDPK-like kinases was increased (Yeh et al., 2007). In similar findings in *O. sativa* plants, CDPK-like kinase was induced in response to the Cr (VI) stress (Trinh et al., 2014). To regulate the production of ROS in plasma membranes, two CDPKs (StCDPK4 and StCDPK5) can phosphorylate NADPH oxidases (Kobayashi et al., 2007). So, it may be concluded that NADPH oxidase and CDPKs may control ROS production during Cr (VI) treatment.

## Conclusions and future research

This review article provides a comprehensive overview of the intricate interplay between Cr and plants, elucidated through cutting-edge omics approaches in systems biology. We have gained insights into the molecular mechanisms governing Cr-plant interactions by integrating genomics, transcriptomics, proteomics, and metallomics data. These insights deepen our understanding of the stress responses and detoxification strategies employed by plants in the presence of Cr and hold immense promise for developing sustainable agricultural practices and phytoremediation tactics for detoxifying Cr-contaminated soils. As we unravel the complexities of this dynamic relationship, it is evident that systems biology will remain indispensable for decoding the fascinating world of Cr-plant interactions. Furthermore, delving into the intricate mechanisms of Cr-plant interactions not only enhances our comprehension of plant stress responses but also opens avenues for innovative biotechnological applications. The exploration of plant-specific genetic pathways and signaling cascades triggered by Cr exposure offers a blueprint for engineering crops with enhanced resistance to metal stress. This knowledge could revolutionize crop breeding programs, leading to the development of resilient varieties capable of thriving in Cr-contaminated environments, ultimately contributing to global food security.

Through the use of omics techniques and systems biology, significant advancements have been achieved in our comprehension of the intricate interaction between Cr and plants. However, a number of knowledge gaps and barriers still exist, necessitating additional research in the years to come. Even though the bulk of studies have concentrated on the immediate effects of Cr exposure, further research is necessary to fully understand how long-term Cr pollution affects plant growth, reproduction, and ecosystem dynamics. For a thorough evaluation of its overall effect on agricultural systems and natural environments, this area of research is essential. Additionally, further study is required to understand how environmental elements, such as soil qualities and climate conditions, affect the response of plants. Moreover, using plants for Cr phytoremediation holds promise, it will be difficult to put this technology into practice in contaminated areas. Innovative methods for boosting phytoremediation efficiency and effectiveness should be explored in further research. Beyond individual plants, there are obvious study gaps when it comes to assessing Cr’s effects on ecosystem services, soil microbial communities, and the larger food web in field trials. Our grasp of this complex relationship and turning it into action will be greatly advanced by continuing multidisciplinary research and collaborative projects.

## Author contributions

FA: Conceptualization, Data curation, Methodology, Resources, Visualization, Writing – original draft, Writing – review & editing. KW: Conceptualization, Investigation, Methodology, Resources, Supervision, Visualization, Writing – original draft, Writing – review & editing. MN: Resources, Supervision, Visualization, Writing – review & editing. PJ: Writing – review & editing. UJ: Resources, Visualization, Writing – review & editing. TA: Conceptualization, Formal Analysis, Investigation, Methodology, Resources, Supervision, Visualization, Writing – original draft, Writing – review & editing. PP: Funding acquisition, Investigation, Project administration, Resources, Supervision, Visualization, Writing – review & editing.
