# Supplementary material for: Systems biology of chromium-plant interaction: insights from omics approaches
Source: Front Plant Sci. 2024 Jan 8;14:1305179. doi: 10.3389/fpls.2023.1305179 (PMC10800501; doi:10.3389/fpls.2023.1305179)
Supplement: Supplementary file 1 [file Table_1.docx]

**Table 1.** Compilation of recent omics studies carried out to understand the response of different plants to Cr stress.

| **Cr source** | **Cr concentration and duration** | **Model plant** | **OMICS strategies induced plant response under HM toxicities** | **Reference** |
| --- | --- | --- | --- | --- |
| K_2_CrO_4_ | 0, 200, and 400 ppm for 25 days | *Helianthus annuus* | 52 and 16 kDa were down-regulated. Five proteins of 60, 42, 47, 49, and 13 kDa were up-regulated to alleviate Cr toxicity | Sardar et al., 2022 |
| K_2_Cr2O_7_ | 100 μmol L^− 1^ for  7 days | *Zea mays* | The proteins PR 1 and 4, Prx5:3, GPX5, abscisic homolog protein, MSD, ferritin 1:6, glyoxalase1:22, and glyoxalase 1:23 were up-regulated, and 5 proteins (peroxidase1, ACC oxidase1, germin 1–1, stress-related proteins, and pathogenesis) were significantly downregulated. | Terzi and Yildiz, 2021 |
| K_2_Cr_2_O_7_ | 1, 5, and 25 mg L^−1^ for 7 days | *Helianthus annuus* | 70% of metabolites involved in the LA metabolic pathway are affected by Cr (VI) stress. | Ibarra et al., 2019 |
| K_2_Cr_2_O_7_ | 200 mg L^−1^; for 6, 12, 24, 48 and 96 h, | *Raphanus sativus* | Enhancement in transcriptional processes like SPLs, MYBs, ERFs, and bZIPs. Expression of these transcription factors aids the plant under Cr stress. | Liu et al. 2015 |
| K_2_CrO_4_ | 50 μM for 24h  50 μM for 1h and 3h | *Oryza sativa* | More transcripts were responsive and involved in cytokinin signaling, ubiquitin–proteasome system pathway, DNA repair, and Cu transportation.  The protein kinase receptors like cytoplasmic kinase MAPK CDPK were significantly upregulated. | Huang et al., 2014 |
| K_2_CrO_4_ | 200 μM for 15–240 min | *Oryza sativa* | Transcripts for two *OsACS* and two *OsACO* genes involved in ACC synthase biosynthesis, which encode key enzymes in ethylene biosynthesis, were strongly induced. | Trinh et al., 2014 |
| K_2_Cr_2_O_7_ | 600 mg L^−1^ for 72 hr | *Raphanus sativus* | 1424 unigenes were up-regulated, and 1561 unigenes were down-regulated and these were involved in the antioxidant system, signal transduction and TFs, transporters, and chelate compound biosynthesis. | Xie et al., 2015 |
| K_2_Cr_2_O_7_ | 100 μM for 72 hr | *Oryza sativa* | Two miRNAs were downregulated under Cr stress.   1. OSA-miR160 (broad-spectrum resistance to fungal and bacterial pathogens). 2. osa-miR1883 (heavy metal-responsive miRNA) is an ATP-binding protein). | Dubey et al., 2020 |
| K_2_Cr_2_O_7_ | 2, 5, 10, 20, 50, 100, 200, 300, 400, 500, 600, 900, 1200, 1500 ppm for 72 h | *Zea mays* | SAM (stressed response protein) is involved in ethylene synthesis during Cr stress. | Labra et al., 2006 |
